# *KRAS* genotyping by digital PCR combined with melting curve analysis

**DOI:** 10.1038/s41598-019-38822-1

**Published:** 2019-02-22

**Authors:** Junko Tanaka, Tatsuo Nakagawa, Akiko Shiratori, Yuzuru Shimazaki, Chihiro Uematsu, Masao Kamahori, Takahide Yokoi, Kunio Harada, Yoshinobu Kohara

**Affiliations:** Center for Technology Innovation - Healthcare, Research & Development Group, Hitachi, Ltd.,1-280, Higashi-koigakubo, Kokubunji-shi, Tokyo 185–8601 Japan

## Abstract

Digital PCR (dPCR) has been developed as a method that can quantify nucleic acids more sensitively than real-time PCR. However, dPCR exhibits large fluctuations in the fluorescence intensity of the compartment, resulting in low accuracy. The main cause is most likely due to insufficient PCR. In this study, we proposed a new method that combines dPCR with melting curve analysis and applied that method to *KRAS* genotyping. Since the melting temperature (Tm) of the PCR product hardly depends on the amplification efficiency, genotyping accuracy is improved by using the Tm value. The results showed that the peaks of the distribution of the Tm values of DNA in the wells were 68.7, 66.3, and 62.6 °C for wild-type *KRAS*, the G12R mutant, and the G12D mutant, respectively, and the standard deviation of the Tm values was 0.2 °C for each genotype. This result indicates that the proposed method is capable of discriminating between the wild-type sequence and the two mutants. To the best of our knowledge, this is the first demonstration of the genotyping of single mutations by combining melting curve analysis and dPCR. The application of this approach could be useful for the quantification and genotyping of cancer-related genes in low-abundance samples.

## Introduction

Circulating tumor DNA (ctDNA) is a liquid biopsy target that may provide an opportunity for noninvasive cancer diagnosis^1–3^. ctDNA refers to small DNA fragments in the blood that are derived from apoptotic and necrotic tumor cells. Normal cell-free DNA (cfDNA) is also released from noncancerous cells, and ctDNA is mixed with normal cfDNA in the blood of cancer patients. The levels of cfDNA in the blood range between 0 and >1,000 ng/ml, with an average of 180 ng/ml^3^. Although cfDNA levels reflect the condition of cancer patients, the levels are generally low, and a very small fraction of cfDNA is derived from tumors. Hence, highly sensitive genetic testing technology that is capable of detecting a trace amount of ctDNA is necessary for the diagnosis of cancer and the monitoring of chemotherapy-resistant mutations.

In genetic diagnosis, quantitative real-time PCR (qPCR) has been widely used for the detection and quantification of specific nucleic acid sequences^4^. qPCR can quantify the initial concentration of a target gene by observing the process of exponentially amplifying a target gene using fluorescently labeled probes or DNA intercalators in real time. Because it generally requires standard curves to determine the initial concentration of the target gene, qPCR is not suitable for absolute quantification. The result can fluctuate over time and across laboratories, and it is not suitable for quantitating very small amounts of samples.

Digital PCR (dPCR) has been developed as a method that can quantify nucleic acids more sensitively than real-time PCR^5–10^. In dPCR, the samples are diluted and divided into many separate compartments. Each compartment contains either one copy or zero copies of the target gene. PCR is performed, and the endpoint fluorescence is measured to determine if the droplet is positive or negative. Then, the target gene in the sample can be digitally counted. Currently, several strategies have been commercialized for dPCR. A droplet-based strategy uses water-in-oil droplets, and PCR is performed in each droplet^9,10^. With this strategy, it is possible to conveniently increase the number of droplets, enabling high-throughput measurements. Another strategy, called BEAMing, uses water-in-oil droplets, and amplicons are coupled to the bead via a biotin-streptavidin linkage after emulsion PCR^7,8^. The beads are recovered by breaking the droplets, hybridized by incubation with fluorescent probes and counted by flow cytometry. A microwell-based strategy uses microwells on the chip to divide the PCR mixture containing sample^5,6^. Since both amplification and fluorescence detection can be performed in the same chip, the measurement scheme is simpler than those of other droplet-based strategies.

**Figure 1:**
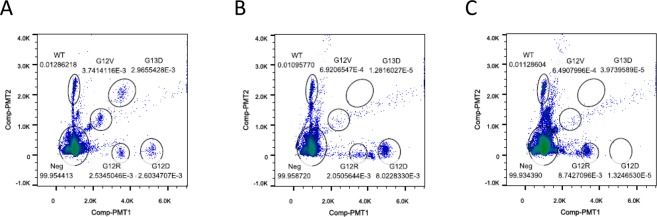
Two-dimensional histogram of conventional dPCR for KRAS genotyping. Multiplex dPCR assays were performed to detect WT KRAS and the four most common KRAS mutations in pancreatic ductal adenocarcinoma: G12D, G12R, G12V and G13D^27^, by using the RainDrop digital PCR system (RainDance Technologies, Billerica, MA), as previously described^26^. (**A**) dPCR plot of the 5-plex assay using a mixture of four types of KRAS mutant genomic DNA reference standards as the DNA template. (**B**) dPCR plot using the genomic DNA reference standard of the G12D mutant as the DNA template. (**C**) dPCR plot using the genomic DNA reference standard of the G12R mutant as the DNA template.

Because dPCR is an endpoint assay, it was conventionally thought that the reaction efficiency of PCR does not significantly affect the measurement result of dPCR. However, as shown in Fig. 1, dPCR exhibits large fluctuations in the fluorescence intensity of the droplets or wells. In the 2-plex assay, the wild-type group and mutant group were able to be separated by the color of the fluorescent dye, even if the groups had a large fluorescence intensity distribution. In the multiplex assay, each group was divided by fluorescence intensity and fluorescent dye color (Fig. 1A). In such a case, a large fluorescence intensity distribution can cause overlap between two groups, resulting in false positives and false negatives (Fig. 1B,C).

Since the main cause of fluctuations in fluorescence intensity is probably due to insufficient PCR in the small partitions, in this study, we have proposed a new measurement method that combines dPCR with melting curve analysis using molecular beacons to solve the above-mentioned problems, and we applied this method to *KRAS* genotyping. The fluorescence intensity of the PCR product-probe hybrid depends on the amount of PCR product, while the melting temperature of the PCR product-probe hybrid is independent of the amount of PCR product and depends on the sequence of the hybridized region. Therefore, the melting temperature is stable regardless of the PCR amplification efficiency. Indeed, several studies have reported the identification of mutations using melting temperatures by high-resolution melting analysis^11–13^. Here, melting curve analysis was performed in small wells, where asymmetric PCR was performed using molecular beacons with hydrophobic stems, which improved the signal-to-background ratio of the melting curves.

## Results

### Strategy for dPCR combined with melting curve analysis

**Figure 2:**
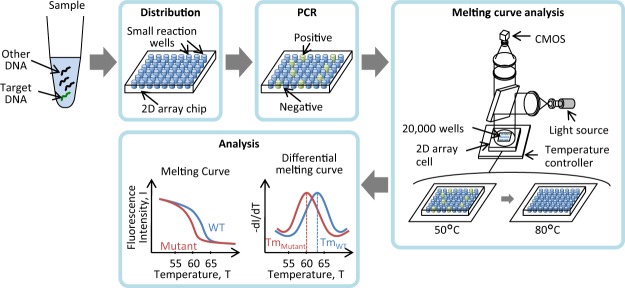
Scheme for dPCR combined with melting curve analysis. The sample is partitioned into many small reaction wells of a 2D-array chip. After PCR, a 2D-array chip is set on the temperature controller, and the fluorescence intensities of the wells are measured with increasing temperature. The melting temperature is calculated from the melting curve.

The scheme for dPCR combined with melting curve analysis is shown in Fig. 2. First, the PCR mixture containing the DNA sample is partitioned into many small reaction wells on a 2D-array chip. Each well contains either one copy or zero copies of the target gene. Second, PCR is performed in each small well. Finally, the 2D-array chip is set on the temperature controller of the prototype dPCR instrument, as shown in Fig. 2, and the fluorescence intensities of the wells are measured with increasing temperature. A melting curve is prepared from the change in fluorescence intensity, and the melting temperature (Tm) is calculated from the differential melting curve.

**Figure 3:**
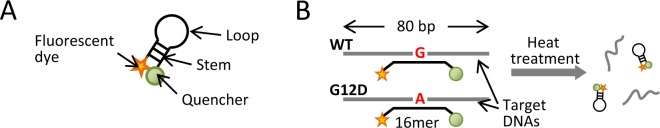
Schematic view of melting curve analysis using a molecular beacon. (**A**) Structure of the molecular beacon. (**B**) Schematic representation of fluorescence signal generation with hybridization of target DNA-specific molecular beacons.

In this study, a molecular beacon^14–16^ was used for both PCR and melting curve analysis, which were performed in the same wells. Although the TaqMan probe is widely used for dPCR, it is not suitable for melting curve analysis because the probe is hydrolyzed by the polymerase during PCR. Molecular beacons have a stem-loop structure, with a fluorescent molecule and quencher at the end (Fig. 3A). Molecular beacons are less likely to be degraded by polymerase than TaqMan probes due to the presence of stems. As shown in Fig. 3B, the free nonhybridized molecular beacon is basically nonfluorescent due to the existence of a stem-loop structure that causes quenching. On the other hand, since the sequence of the loop portion is complementary to the target sequence, the molecular beacon becomes strongly fluorescent when hybridized with its target at a low temperature. As the temperature increases, the complex of the molecular beacon and target dissociates, and the molecular beacon becomes a random-coil structure like the TaqMan probe, which has weak fluorescence.

### Optimization of conditions for PCR and melting curve analysis

**Figure 4:**
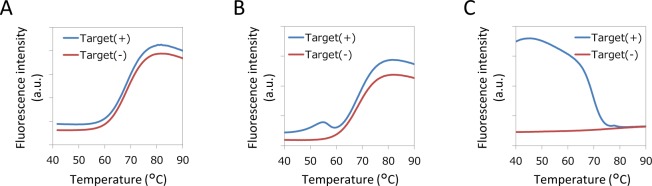
Comparison of the melting curves of probe-target hybrids under various conditions. (**A**) Symmetric PCR. (**B**) Asymmetric PCR. (**C**) Asymmetric PCR using molecular beacons with hydrophobic stems.

To optimize the performance of both PCR and melting curve analysis, we propose to use asymmetric PCR and a molecular beacon with a hydrophobic stem. The asymmetric PCR is necessary to obtain more single-stranded amplicons complementary to the molecular beacon probe and fewer noncomplementary single-stranded amplicons. Figure 4 shows the results of melting curve analysis performed in solution after amplifying the target sequence, the *KRAS* gene. With symmetric PCR, the melting curve of the probe-target hybrid could not be observed (Fig. 4A). In the reaction solution, in which symmetric PCR was performed, it is expected that the complementary strands of the amplicon preferentially hybridize with each other, and the proportion of the probe-target hybrid is extremely small. However, after performing asymmetric PCR^14–17^, a single-stranded amplicon complementary to the probe is generated in excessive amounts (Supplementary Fig. S1), and a melting curve of the probe-target hybrid can be observed (Fig. 4B). However, even when asymmetric PCR was performed, the change in the fluorescence intensity of the probe itself was larger than that of the probe-target hybrid due to the temperature change because the amount of amplified ssDNA is small compared to the total amount of probe added to the PCR mixture. Due to the background change, the melting curve analysis was still difficult.

To improve the signal-to-background ratio of the melting curves, we utilized a molecular beacon with a hydrophobic stem^18,19^. The use of molecular beacons with hydrophobic stems can maintain background fluorescence at a constant value, even at high temperatures (Fig. 4C), because hydrophobic stems are formed in unbound probes in a temperature-independent manner. The change in the fluorescence intensity of the PCR solution when using molecular beacons with hydrophobic stems during the measurement was one-tenth of that obtained when using molecular beacons without hydrophobic stems.

The use of asymmetric PCR and molecular beacons with hydrophobic stems significantly improved the signal-to-background ratio of the melting curve compared to that of the melting curve obtained under the original conditions. We applied the optimal conditions for PCR and melting curve analysis to dPCR.

### dPCR with melting curve analysis

**Figure 5:**
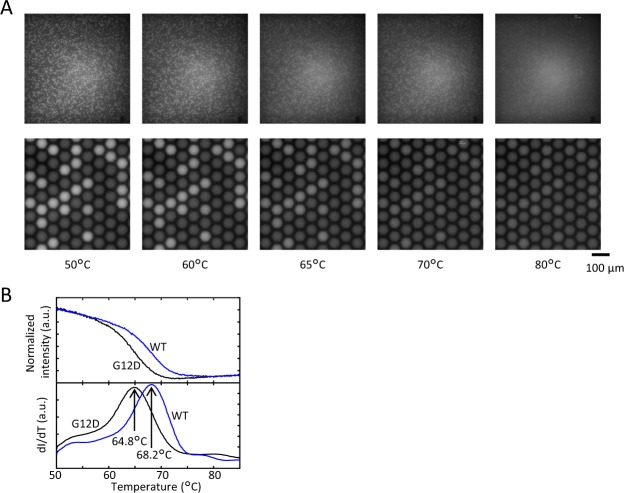
Fluorescence image of a 2D-array chip and the melting curve measured from changes in the fluorescence intensity of the wells. (**A**) Fluorescence image of the 2D-array chip at each temperature. During controlled heating, the hybrid of the target and the probe dissociates, and fluorescence is lost. (**B**) Melting curve of WT *KRAS* and the G12D mutants derived from the change in fluorescence intensity of a well on a 2D-array chip.

To demonstrate genotyping by Tm values, PCR and melting curve analysis for the WT and G12D mutant of *KRAS* were performed by using molecular beacons with the same dye, FAM. The PCR mixture containing equal amounts of the WT and G12D mutant of *KRAS* was partitioned into 20,000 reaction wells on a 2D-array chip. After PCR, the fluorescence of each well was analyzed with increasing temperature. As shown in Fig. 5A and Supplementary Video, there were bright wells and dark wells at 50 °C. The bright wells were considered ‘positive’ wells and contained the *KRAS* DNA, whereas the dark wells were ‘negative’ wells and did not contain the *KRAS* DNA. When the temperature of the 2D-array chip was increased, the fluorescence intensity of the bright wells decreased, and the fluorescence intensity of all the wells became similarly dark at 80 °C. Figure 5B shows that, as expected, the fluorescence intensity of each well decreased as the temperature increased in a target-dependent manner, indicating the existence of different Tm values for each target that could be derived from the differential melting curve.

**Figure 6:**
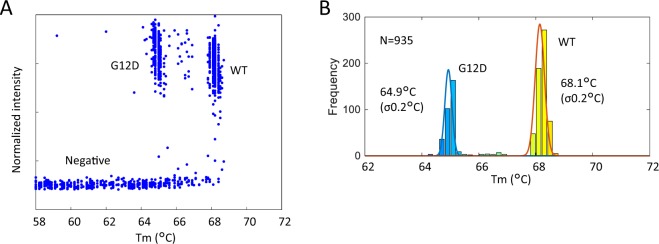
Genotyping of *KRAS* mutation with a 2-plex assay. (**A**) The relationship between Tm and the normalized intensity of each well. The groups (‘negative’ wells, wells containing WT DNA, and wells containing G12D mutant DNA) were divided by Tm and normalized intensity. (**B**) Tm histogram of ‘positive’ wells.

To separate ‘positive’ and ‘negative’ wells, we used the differences in fluorescence intensity among the wells. In Fig. 6A, which shows the relationship between the Tm value and normalized fluorescence intensity, positive wells and negative wells can be easily distinguished based on normalized fluorescence intensities. The fluorescence intensity of the negative wells was weaker than that of the positive wells because the negative wells contained no probe-target hybrid. Therefore, the differential melting curves had no clear peak, and the Tm values varied greatly. The histogram of ‘positive’ wells reveals that the peaks of the distribution of the Tm values of positive wells were 68.1 °C and 64.9 °C for WT and the G12D mutant, respectively, with the standard deviation of the Tm value being 0.2 °C for both genotypes (Fig. 6B). There are several dots outside of the WT and G12D groups in Fig. 6A, and there is a small peak between the peak of the WT and the peak of the G12D mutant in Fig. 6B. These plots and small peaks are presumed to contain both WT DNA and G12D mutant DNA in one well. It was presumed that the detected number of WT was greater than the detected number of G12D, because the number of molecules could not be completely controlled, when WT and PCR amplicon of G12D mutant were individually prepared and then mixed in equal amounts each. The *KRAS* G12D mutant has a single nucleotide mutation in codon-12 of exon-2 that induces replacement of the GGT sequence (encoding glycine) by the GAT sequence (encoding aspartic acid)^20^. Hence, the GC content of the *KRAS* G12D mutant sequence is lower than that of the WT sequence, and the Tm value of the G12D mutant is observed to be low. The relative relationship of the Tm values of the WT and G12D mutants was consistent with the results of qPCR (Supplementary Table S1). Note that the Tm values of dPCR are slightly lower than those of qPCR because the temperature shown in the result of dPCR is the setting value of the temperature controller, which is slightly different from the temperature of the 2D-array chip. The measured difference in Tm values between WT and the G12D mutant is 3.2 °C, which is caused by the single nucleotide mutation. This difference, which is equal to 16 times the standard deviation, is large enough to detect a small amount of the mutant. According to the simulation results, assuming that the distribution of Tm values follows a normal distribution, it is necessary that the difference in Tm values is 5 times the standard deviation to detect mutants present at an abundance of 1% (data not shown). Therefore, this result indicated that our proposed methods probably have enough accuracy for detecting <1% single nucleotide variants.

### Multiplex assay for *KRAS* genotyping

**Figure 7:**
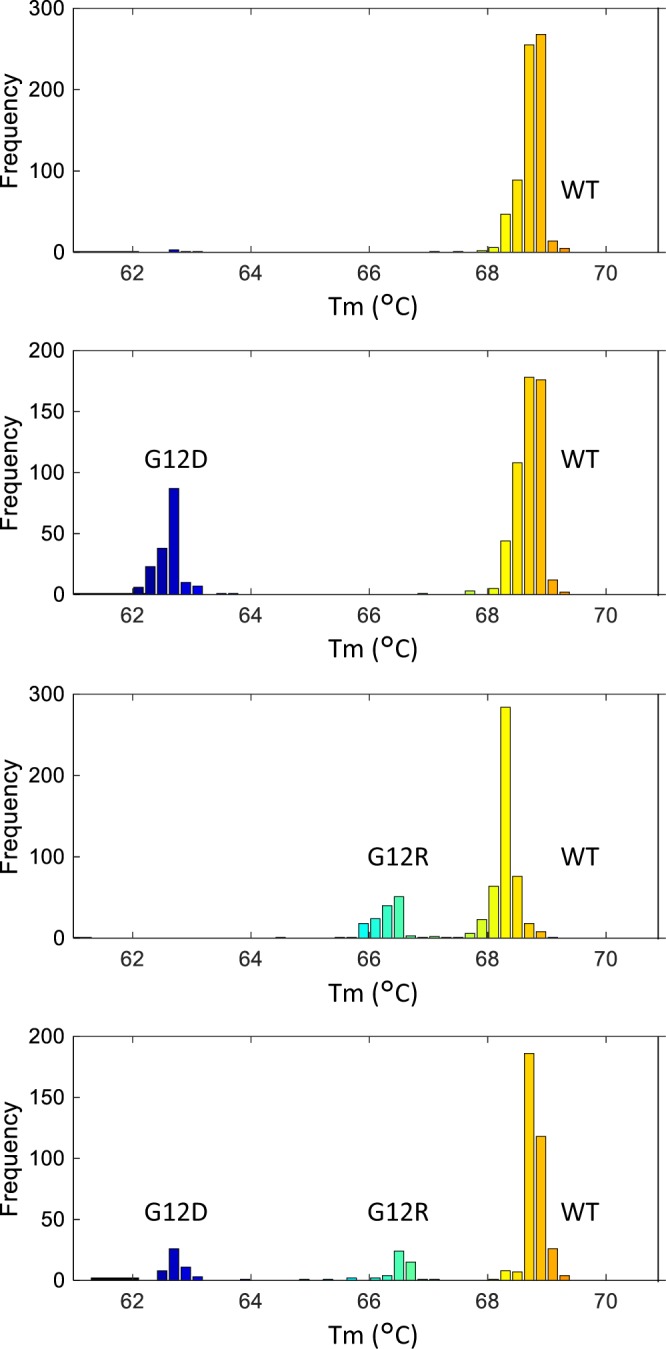
Genotyping of *KRAS* mutations with a 3-plex assay. 3-plex assays were performed to detect WT KRAS and two KRAS mutations: G12D and G12R.

A multiplex panel assay for *KRAS* genotyping was performed by mixing mutation-specific molecular beacons with a WT-specific molecular beacon and a single pair of PCR primers. Genomic DNA standards for the *KRAS* mutation were used for the multiplex assay. As shown in Fig. 7, the melting temperature of the probe was tuned to discriminate between wells containing WT *KRAS* DNA and wells containing DNA with a unique *KRAS* mutation. The histogram shows that the peaks of the distribution of the Tm values of the positive wells were 68.7 °C, 66.3 °C, and 62.6 °C for WT, the G12R mutant, and the G12D mutant, respectively (Fig. 7). Thus, we have successfully demonstrated the use of the 3-plex panel for *KRAS* genotyping using dPCR with melting curve analysis.

The Tm value of G12D was observed to be 2.3 °C lower in the 3-plex assay than in the 2-plex assay. This difference occurs because the sequence of the probe used for G12D differs between the two assays. To increase the difference between the Tm values of G12D and G12R, the sequence of the probe for G12D used in the 3-plex assay is one base shorter than the probe used in the 2-plex assay. In this way, the Tm value can be controlled by designing the sequence of the probe according to the type of assay.

## Discussion

This report provides the first evidence that genotyping of a single nucleotide mutation of a cancer-related gene can be realized by combining melting curve analysis and dPCR. Previously, pathogen genotyping has been reported by combining melting curve analysis and dPCR^21^. In this previous study, since melting curve analysis was performed using a DNA intercalator, the Tm value was determined by the sequence of the entire amplicon. For example, the calculated Tm values of *KRAS* amplified with the same primer used in this report are 90.8 and 90.3 °C for WT and the G12D mutant, respectively, using the nearest-neighbor model^22^. The difference in Tm values due to a single nucleotide mutation is 0.5 °C. Therefore, to detect mutants present at an abundance of 1% by melting curve analysis using a DNA intercalator, a device with a standard deviation for Tm values of less than 0.1 °C is required. In this study, by using a molecular beacon, the difference in Tm values due to a single nucleotide mutation becomes larger than that using a DNA intercalator, and detection becomes feasible. For cancer diagnosis, there are many opportunities for the detection of single nucleotide mutations, such as the prediction of recurrence and the monitoring of drug resistance genes for the selection of molecular target drugs. This study shows the possibility of detecting a single mutation with both high accuracy and high sensitivity, even with a sample with low abundance, such as ctDNA.

In this study, the probe was labeled with a single-color fluorescent dye, and a multiplex assay was performed. For cancer diagnosis, it is desirable to examine multiple mutations of multiple cancer-related genes that are suspected to be affected at the same time. It is thought that this multiplicity can be further increased by increasing the types of fluorescent dyes used for labeling. Conventionally, the multiplex dPCR assay was performed with the color and intensity of fluorescent dye^23–26^, but the fluctuations in the fluorescence intensity were large, and it was difficult to separate clusters of each target. In particular, ctDNA and DNA from the FFPE sample were damaged and fragmented, so these samples are presumably difficult to multiplex due to the difficulty of DNA amplification by PCR. A multiplex assay performed using the color of the fluorescent dye and the Tm value is expected to be highly effective in the measurement of samples such as ctDNA and FFPE samples, which were difficult to analyze in conventional studies.

## Methods

### Preparation of DNA templates

The *KRAS* gene was amplified using genomic DNA extracted from HCT116 p21 (+) as a template and cloned into the pCR2.1-TOPO vector (Thermo Fisher Scientific, Waltham, MA). The G12D mutation was introduced by PCR-based site-directed mutagenesis using genomic DNA extracted from HCT116 p21 (+) as a template, and the mutant was cloned into the pCR2.1-TOPO vector. Wild-type (WT) and G12D-mutant *KRAS* were amplified from plasmids using a forward primer (5′-GTAAAACGACGGCCAG-3′) and a reverse primer (5′-CAGGAAACAGCTATGAC-3′) and purified using a QIAquick PCR Purification Kit (QIAGEN, Hilden, Germany). PCR amplicons were used as the DNA template in real-time PCR and digital PCR.

*KRAS* mutant genomic DNA reference standards (Horizon Diagnostics, Cambridge, UK) were also used as DNA templates in digital PCR.

### Real-time PCR

Asymmetric PCR with molecular beacons to detect *KRAS* mutations was performed by real-time PCR. Molecular beacons with hydrophobic moieties in the stem, dye at the 5′ end and quencher at the 3′ end were synthesized (PentaBase, Odense, Denmark). The sequences of the employed primers and molecular beacons are described in Table 1. Samples for real-time PCR were prepared by mixing 10 μl of TaqMan Genotyping Master Mix (Thermo Fisher Scientific), 0.25 μM forward primer, 0.5 μM reverse primer, 0.5 μM each molecular beacon for WT and G12D-mutant *KRAS*, and the DNA template containing approximately 2 × 10^7^ copies of the WT *KRAS* gene in a final reaction volume of 10 μl. Amplification and melting curve analysis were performed with a QuantStudio 12 K Flex real-time PCR system (Thermo Fisher Scientific). Amplification was carried out as follows: 10 min at 95 °C and 60 cycles of 15 s at 95 °C and 30 s at 60 °C. Melting curve analysis was carried out by increasing the temperature from 40 °C to 95 °C at a rate of 3.0 °C/min.

**Table 1:** List of primers and probes.

Name	Sequence	Modification
Forward primer	5′-AGGCCTGCTGAAAATGACTGAATAT-3′	—
Reverse primer	5′-GCTGTATCGTCAAGGCACTCTT-3′	—
Probe for WT	5′-XXXXTTGGAGCTGGTGGCGTXXXX-3′	5′FAM, 3′BHQ-1
Probe for G12D (for 2-plex assay)	5′-XXXXTTGGAGCTGATGGCGTXXXX-3′	5′FAM, 3′BHQ-1
Probe for G12D (for 3-plex assay)	5′-XXXXTGGAGCTGATGGCGTXXXX-3’	5′FAM, 3′BHQ-1
Probe for G12R	5′-XXXXTTGGAGCTCGTGGCGTXXXX-3′	5′FAM, 3′BHQ-1

X indicates a hydrophobic DNA analog.

### dPCR in the wells

dPCR combined with asymmetric PCR was performed using the molecular beacons in the wells. The sequences of the employed primers and molecular beacons are described in Table 1. Samples for dPCR were prepared by mixing 1x QuantStudio 3D Digital PCR Master Mix v2 (Thermo Fisher Scientific), 0.25 μM forward primer, 0.5 μM reverse primer, 0.5 μM each of molecular beacons for the WT, G12R-mutant and G12D-mutant *KRAS*, and DNA template containing approximately 1 × 10^3^ copies of the *KRAS* gene in a final reaction volume of 15 μl. The samples were divided into 20,000 wells on a QuantStudio 3D Digital PCR 20 K Chip (Thermo Fisher Scientific). Amplification in the wells was carried out by the thermal cycler as follows: 10 min at 95 °C and 60 cycles of 15 s at 95 °C and 75 s at 60 °C.

### Melting analysis by microscopic imaging

The melting analysis of the wells in the 2D-array chip was performed by microscopy with a thermal stage. The excitation light generated by a 490 nm blue LED (Thorlabs, Newton, NJ) was focused on the inside 2D-array chip on the thermal stage after transmitting the light through a light guide, three 490 nm bandpass filters, a 500 nm dichroic filter and an objective. The focal spot size was 1 cm × 1 cm. The dyes of the molecular beacon were excited by the excitation light, and the fluorescence emitted from fluorescent droplets was collected by the same objective and transmitted through the same dichroic filter, a 520 nm long pass filter, and three 520 nm bandpass filters. Fluorescence images of the droplets in the 2D-array chip were detected by an ORCA-Flash4.0 V3 digital CMOS camera (Hamamatsu Photonics, Hamamatsu, Japan) while increasing the temperature of the thermal stage from 50 °C to 85 °C at a rate of 3.0 °C/min.

## Supplementary information

Supplementary video

Supplementary information

## Data Availability

All supporting data are available at Supplementary Data.
