# Supplementary material for: KRAS genotyping by digital PCR combined with melting curve analysis
Source: Sci Rep. 2019 Feb 22;9:2626. doi: 10.1038/s41598-019-38822-1 (PMC6384904; doi:10.1038/s41598-019-38822-1)
Supplement: Supplementary file 2 — Supplementary information [file 41598_2019_38822_MOESM2_ESM.pdf]

Supplementary information for

KRAS genotyping by digital PCR combined with melting curve analysis

Junko Tanaka, Tatsuo Nakagawa, Akiko Shiratori, Yuzuru Shimazaki, Chihiro Uematsu, Masao Kamahori, Takahide Yokoi, Kunio Harada, and Yoshinobu Kohara

This file includes:

- Supplementary Table S1.
- Supplementary Figure S1.

Supplementary Table S1. Tm values measured by qPCR.

Probe name	Tm values (°C)
**Probe for WT**	70.8
**Probe for G12D (for 2-plex assay)**	67.7
**Probe for G12D (for 3-plex assay)**	65.7
**Probe for G12R**	69.9

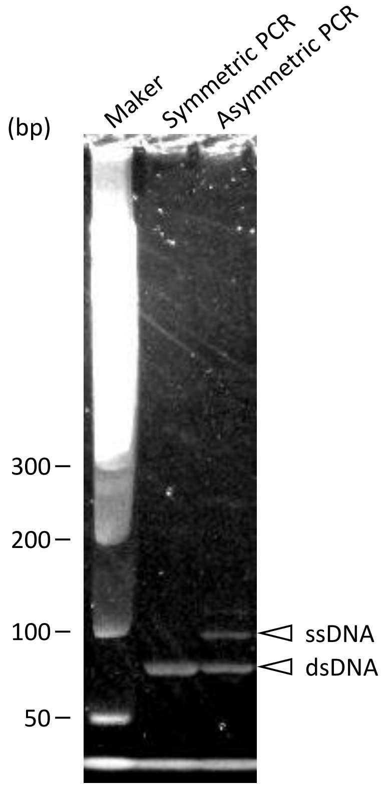

Supplementary Figure S1. The amplified DNA was amplified by symmetric PCR and asymmetric PCR. The PCR products were resolved by 10% polyacrylamide gel electrophoresis (PAGE) and detected by SYBR Gold staining. The full-length ssDNA of the amplicon complementary to the probe was synthesized and analyzed by PAGE as a control. The DNA amplified by asymmetric PCR was separated into two bands; one band was observed at the same position as the DNA amplified by symmetric PCR, and the other band was observed at the same position as the synthetic ssDNA complementary to the probe. From the results of PAGE, it was confirmed that asymmetric PCR amplified an ssDNA complementary to the probe.
